# Favorable effects of dill tablets and *Ocimum basilicum *L. extract on learning, memory, and hippocampal fatty acid composition in hypercholesterolemic rats

**DOI:** 10.22038/ijbms.2021.49013.11230

**Published:** 2021-03

**Authors:** Neda Heshami, Soheila Mohammadali, Alireza Komaki, Heidar Tayebinia, Jamshid Karimi, Ebrahim Abbasi Oshaghi, Mohammad Hashemnia, Iraj Khodadadi

**Affiliations:** 1 Department of Clinical Biochemistry, Faculty of Medicine, Hamadan University of Medical Sciences, Hamadan, Iran; 2 Neurophysiology Research Center, Hamadan University of Medical Sciences, Hamadan, Iran; 3 Department of Pathobiology, Faculty of Veterinary Medicine, Razi University, Kermanshah, Iran; 4 Nutrition Health Research Center, Hamadan University of Medical Sciences, Hamadan, Iran

**Keywords:** Alzheimer’s disease, Dill, Hypercholesterolemia, Learning, Memory, Ocimum

## Abstract

**Objective(s):**

Hypercholesterolemia is correlated with brain amyloid-β (Aβ) deposition and impaired cognitive functions and contributes to Alzheimer’s disease. Effects of cholesterol-lowering dill tablets and aqueous extract of *Ocimum basilicum *L*.* (basil) on learning and memory and hippocampus fatty acid composition were examined. mRNA levels of the genes involved in cholesterol homeostasis were also determined in high-cholesterol diet (HCD) fed rats.

**Materials and Methods:**

Forty male Wistar rats were allocated to 4 groups: rats fed chow diet (C); rats fed high-cholesterol (2%) diet (HCD); rats treated with HCD+300 mg/kg dill tablets (HCD+Dill); and finally, rats fed HCD and treated with 400 mg/kg basil aqueous extract (HCD+basil). Treatment was carried out for 16 weeks. Hippocampus Aβ(1-42) level was determined. Spatial and passive avoidance tests were used to examine cognitive functions. Hippocampal FA composition was assessed by gas chromatography. Basil aqueous extract was analyzed by GC-double mass spectroscopy (GC-MS/MS) and expression of LXR-α, LXR-β, and ABCA1 genes was assessed by qRT-PCR.

**Results:**

Dill tablets and basil extract remarkably ameliorated serum cholesterol (*P*<0.001), retarded hippocampal accumulation of Aβ, and attenuated HCD-induced memory impairment. Hippocampus FA composition did not change but serum cholesterol was found positively correlated with hippocampus Aβ(1-42) (*P*<0.001), total n 6 PUFA (*P*=0.013), and Aβ(1-42) showed correlation with the ratio of n6 to n3 PUFA. At least 70 components were identified in basil aqueous extract.

**Conclusion:**

Dill tablets and aqueous extract of basil attenuated the hypercholesterolemia-induced memory impairment by lowering serum cholesterol and hippocampus amyloid deposits, and probably beneficial in AD adjuvant therapy.

## Introduction

High-fat and high cholesterol diets increase the prevalence of neurocognitive disorders such as Alzheimer’s disease (1). Previous studies have shown that increased plasma cholesterol concentration is associated with AD and imbalance in homeostasis of cholesterol metabolism leads to neuronal dysfunction and formation of Amyloid β (Aβ) plaque in the brain, where Aβ deposit plays a key role in neuronal damage in AD. Both generation of Aβ in the brain and its clearance are regulated by cholesterol (2). In subjects over 85 years of age, AD affects nearly 40% of individuals and is characterized by progressive cognitive deficits and permanent disability of memory (3).

The brain possesses 23% of total body cholesterol (4), a vital compartment of the eukaryotic cell membranes that impacts membrane fluidity and contributes to nearly all aspects of cellular structure and function. A cholesterol-enriched diet alters hippocampal morphology and impairs cognitive functions in an aged population (5). Though association of hypercholesterolemia and impairment in learning and memory has previously been shown, the exact mechanisms correlating cholesterol and cognitive functions are poorly comprehended. 

Herbal plants contain various natural products that play crucial pharmacological roles in the treatment of different types of diseases. The World Health Organization (WHO) has estimated that herbal medicines especially plant extracts are being used by approximately 80% of the worldwide population for their primary treatment needs (6). In the same way, the use of herbal medicine has been considered for lowering serum cholesterol as reviewed by El-Tantawy and Termaz (6).* Ocimum basilicum *L*.* (basil) and *Anethum graveolens *L. (dill) are culinary herbs that are widely cultivated in almost all countries and traditionally have been used to lower serum lipid contents (7, 8). A study by Amrani *et al*. reported a strong hypolipidemic effect for basil in hyperlipidemia-induced animals (9). Likewise, hypolipidemic and hypocholesterolemic effects of* A. graveolens* extract (10) have been attributed to its flavonoid and phenolic contents (8). Therefore, based on their cholesterol lowering-properties it is hypothesized that consumption of dill tablets and *O. basilicum* aqueous extract may prevent unpleasant effects of HCD on cognitive functions in rats. 

Apart from the role of cholesterol, the proper functioning of the brain is affected by its fatty acid (FA) content and composition. Although there are several lines of evidence reporting neurodegenerative and neuroprotective properties for saturated and unsaturated FAs, respectively, the epidemiological literature show that FA composition of different anatomical areas of the brain is seemingly inconsistent (11). In addition, there is no available convincing evidence about the FA composition of the hippocampus in AD. 

Therefore, this study investigated the possible beneficial effects of dill tablets and *O. basilicum* extract on hippocampal Aβ deposition and intellectual activities including learning and memory. The effects of dill tablets and *O. basilicum* extract were also investigated on the FA composition of the hippocampus in the HCD-induced model of AD in rats. Moreover, the expression of cholesterol metabolism-related genes including ABCA1, LXRα, and LXRβ was quantified in hippocampus tissues. 

## Materials and Methods

Aerial parts of the *O. basilicum* L. plant were obtained from local stores. Plants were identified in Medicinal Plants Research Center (School of Pharmacy, Hamadan University of Medical Sciences, Hamadan, Iran), received MPRC-445 identity approval code, and the name of the plant was checked with http://www.theplantlist.org. The plant was then dried, grounded to soft powder, dissolved in distilled water at 40 °C with gentle shaking for 48 hr in a dark container. Finally, after filtration, the extract was dried and stored at -20 °C. Dill tablets were purchased as a common pharmaceutical product of Iran Darouk Company from a local pharmacy.

***Animals***

Forty male Wistar rats weighing 180–200 g were accommodated, two per cage, in a temperature-controlled room under natural light/dark cycle. Animals were provided *ad libitum* access to water and food throughout the experiment. The study received University Ethics Committee approval, and the National Institutes of Health Guide for the Care and Use of Laboratory Animals (1996) was followed in all experiments and animal care procedures. The minimum number of animals was used, safety procedures were complied with in all mandatory experimental works, and suffering of animals was minimized in all experiments. 

After acclimatization of animals with regular chow diet for one week, every 10 rats were randomly allocated into a group as: (C): Control rats receiving chow diet, (HCD): rats fed high-cholesterol (%2) diet, (HCD+Dill): rats fed HCD and receiving dill tablets (300 mg/kg), and (HCD+basil): rats fed high-cholesterol (%2) diet plus 400 mg/kg aqueous extract of basil. The concentration of dill tablets (300 mg/kg) which was used in the present study was based on the previously described optimal concentration (8, 10) whereas the 400 mg/kg concentration of basil was considered as effective concentration, as previously reported (12).

Basil extract and dill tablets were daily given to animals by gavage for 16 weeks. Shuttle box and Morris water maze experiments were done in the last two weeks of the treatment.

***Composition of HCD***

Control chow diet was a standard laboratory diet for rodents (Behparvar, Iran) containing protein (21%), fat (3.69%), carbohydrates (32.5%), as well as crude fiber (5.5%). To prepare a high-cholesterol diet, the standard chow diet was mixed with cholesterol (2%) and cholic acid at a concentration of 0.5% (Sigma-Aldrich Co. Ltd., UK) and stored at -20 °C (10).

***Learning and memory tests***

In this study, spatial memory and passive avoidance learning (acquisition and retention) were determined by Morris Water Maze (MWM) behavioral task and shuttle box tests, respectively.

***Spatial memory test (MWM task)***

The MWM task was carried out in a circular water pool (R=180 cm and H=60 cm) filled to a depth of 25 cm. The task was performed in dim illumination and a soundproof room with some visual sensory cues to help animals for accurate navigation. The pool consisted of four quadrants and an invisible 10×10 cm^2^ square platform was located one centimeter underneath the water surface in the target quadrant. The training step was carried out for 4 days with each day of training including 2 blocks with 4 trials and lasting for 60 sec in each trial. The gap between every two trial was 30 sec with 30 min resting time between two consecutively running blocks. The escape latency was recorded with a Nikon DSLR videography camera (Nikon Corporation, Japan) which was located above the pool and connected directly to a computer. On day 5, the platform was removed and memory retention was determined as the time spent by animals in the target quadrant in a 60-sec probe trial.

***Passive avoidance learning test (shuttle box)***

The shuttle box was made of two compartments including a bright transparent (20 × 20 × 30 cm) and a same size dark compartment with opaque walls. In both compartments, the grid floors consisted of stainless-steel rods with one centimeter space between rods. A constant current AC electric shock generator was used to electrify the floor of the dark compartment. Two compartments were separated by a 6 × 8 cm rectangular opening hatch with an automotive opaque guillotine door. 

Each rat was habituated to the apparatus in two trials. First, the rat was positioned in the bright section back onto the door and after 30 sec the guillotine door was opened. Since animals have a tendency toward darker places by instinct, rats attempted to leave the lighted section. Upon the entrance to the dark section, the guillotine door was shut and after 30 sec of habituation, the rat was transferred to the home cage. The habituation trial was repeated after half an hour with a similar interval to the first acquisition trial. Step-through latency (STLa) was considered as the latency of entrance to the dark part of the box. Thereafter, the sliding door was closed and a non-lethal electrical shock was administered through the grid floor for 1.5 sec. The rat was transferred into the cage and the test was repeated after 5 min by re-administration of electrical shock once the rat entered the dark section. Upon accomplishment of the training, the rat was kept in the light section for 2 min and the number of the entrances to the dark section was counted.

Eventually, the long-term memory was tested after a 24 hr delay period. The rat was positioned in the lighted section, the sliding door between two compartments lifted up, and the data for step-through latency (STLr) as representative of suppressed instinct preference to the darker places, as well as the mean time spent by the rat in the dark (TDC) section, were recorded for 5 min without administration of electric shock.

***Sample collection***

After completing the behavioral studies, the overnight fasting rats were anesthetized and sacrificed. Blood samples were collected from the vena cava and serum samples were separated and stored at -20 °C. The hippocampus tissues were dissected midsagittally and immediately immersed in liquid nitrogen. For histopathology examination, the whole brains were collected and fixed in formalin. 

***Histopathology analysis***

Brain tissues were fixed in 10% formalin, dehydrated by alcohol, and embedded in paraffin. Then, tissue sections (5 µm in thickness) were prepared, stained with Hematoxylin and Eosin and Congo red, and analyzed under light microscopy.

***Determination of serum lipid profile***

Serum lipid profile including triglycerides (TG), total cholesterol, and cholesterol contents of LDL and HDL were assessed by commercial enzymatic kits (Pars Azmoon, Tehran, Iran).

***Measurement of hippocampal amyloid β(1-42) content***

The left section of the hippocampus was homogenized in lysis buffer and used for determination of Aβ level. Amyloid β(1-42) content was quantified using a commercial ELISA kit (BioLegend Co., USA), based on the manufacturer’s protocol. 

***Identifying fatty acid composition of hippocampus tissues***

Briefly, total lipids were isolated by chloroform and methanol from hippocampal tissue (right section) samples. Nonadecanoic acid (Sigma-Aldrich Co. Ltd., UK), as internal standard, was added (100 µg) to each sample before the total FA extraction, and extracted lipids were dried using a flow of nitrogen gas. Next, FAs were trans-esterified to fatty acyl methyl esters (FAMEs) by saponification process using 20% BF3/methanol and n-hexane. Fatty acyl methyl esters were then isolated with n-hexane (13) and separated by VARIAN CP-3800 gas chromatography (Varian Inc., USA) supplied with a 60 m × 0.25 mm i.d. HP-88 column (Agilent Technologies Inc., USA) and flame ionization detector (FID). The temperature for injector and FID was programmed at 280 °C. The carrier gas (Helium) at 1.2 ml/min flow rate was used and temperature from 160 °C to 220 °C rising 3 °C per min was programmed. The gas chromatograph was calibrated using a mixture of 37 fatty acyl methyl esters as an external standard and the split ratio was set at 10:1. The chromatograms were identified by comparison of the relative retention time of each peak with chromatogram of standards. Besides, a well-identified peak of nonadecanoate (MeC19:0) as internal standard was individually detected confirming no interference of internal standard peak and rat hippocampus chromatogram. 

***Quantification of mRNA levels by qRT-PCR***

The expression levels of hippocampal ABCA1, LXR-α, and LXR-β genes were determined by quantitative real-time PCR. Total RNA was isolated from left hippocampus tissues and its concentration and quality were determined using NanoDrop^TM^ (Thermo Fisher Scientific, USA) and agarose gel electrophoresis, respectively. The synthesis of cDNA was carried out by reverse transcription of 500 ng of total RNA using HyperScriptTM Reverse Transcriptase cDNA (GeneAll Biotechnology Co. Ltd., Korea). The Ampliqon Master Mix and a Roche light cycler 96 (Roche Diagnostics, Germany) were used to conduct Real-time PCR. The sequences of primers were as: forward: 5’-TTCCGTCCTCCTTGTCATCTCTG-3’ and reverse: 5’-GGTCATCATCACTTTGGTCCTTGG-3’ for ABCA1; forward: 5’-TCTCCTGACTCTGCAACGGA-3’ and reverse: 5’-CCAGTTGATTGGGGCATCCT-3’ for LXR-α; forward: 5’-ACGCTACAACCACGAGACAG-3’ and reverse: 5’-GCCCGAGAGAACTCAAAGATG-3’ for LXR-β; and forward: 5’-CCCGCGAGTACAACCTTCT-3’ and reverse: 5’-CGTCATCCATGGCGAACT-3’ for β-actin as control. Relative gene expressions (fold changes) were finally calculated by the Livak formula (2^− Ct^) and normalized to the expression of β-actin.

***Analysis of basil aqueous extract by GC-MS/MS***

The choice of menstruum in herbal extraction usually depends on the type of plant and the end-use. Semipolar or lipophilic solvent extracts are commonly suitable for identification and isolation of secondary compounds of the plants. In contrast, herbalists mostly use plant water decoction or plant aqueous extracts to pharmacologically test a traditionally used phytomedicine or for clinical treatments. Additionally, in using herbs as dietary supplements the microenvironment is aqueous rather than lipophilic therefore aqueous extract of *O. basilicum *L. was used in this study. 

Aqueous extract of basil was analyzed using 7890B Agilent gas chromatography-Mass/Mass spectroscopy (Agilent Technologies, Santa Clara, CA-USA). However, due to the inapplicability of analyzing aqueous samples by GC-MS/MS, chemical components of the basil extract were extracted using various HPLC grade solvents such as 2-propanol, toluene, n-hexane, methanol, and acetonitrile before application to GC-MS/MS apparatus. Briefly, one gram of dried basil extract powder was solubilized in 10 ml organic solvent (methanol), centrifuged, and the supernatant was separated. Then, 9 ml of either of the above-mentioned solvents (e.g. acetonitrile) was added to the precipitant, agitated for 10 min, and centrifuged to collect the supernatant. Next, one microliter of the collected extract (e.g., acetonitrile extract) was injected into the GC. The data were displayed on a 30 m long, 0.25 mm ID, and 0.25 μm thick HP-5MS column. A steady and continuous stream (one milliliter per min) of pure helium gas was applied as the carrier gas. GC oven temperature started from 75 °C and after 4 min holding time, the temperature increased up to 120 °C inclining 25 °C per min and then up to 300 °C with 5 °C increasing per min with 10 min final holding at 300 °C. The 250 °C temperature was applied for injector and detector and the mass range was scanned between 50 and 550 amu. Components of basil extract were identified by comparing the spectrum of any unknown component with the spectrum of known components from the National Institute of Standard and Technology (NIST) database. The relative percentages of components were obtained by comparing the average of correspondent peak areas to the total peak area. 

***Statistical analysis***

Statistical analysis was carried out using the SPSS 16 software (SPSS Inc., Chicago-USA). One-way ANOVA followed by the *post-hoc* Tukey test was used to compare differences between groups, whereas the results of learning and memory experiments were analyzed with two-way ANOVA followed by Bonferroni’s *post-hoc* test. Pearson correlation coefficient (r) was applied to determine the correlation between FA composition and other variables. Data were represented as mean±SEM and *P*<0.05 showed statistical significance.

## Results

***Dill tablets and basil extract favorably modified serum lipid profile***

The bodyweight of animals was recorded in all groups throughout the study. As shown in Table 1, bodyweight did not significantly differ between groups on the start day or at the end of the experiments. 

Data analysis showed (Table 1) no significant difference in the serum TG, total cholesterol, LDL-C, and HDL-C between groups at the beginning of the study. In contrast, on the last day serum cholesterol and LDL-C (but not TG and HDL-C) significantly differed between groups. The results also showed that serum total cholesterol was markedly increased in HCD-fed rats whereas treatment with Dill or basil extract (HCD+Dill and HCD+basil groups) strongly lowered cholesterol levels. Treatment of the rats with basil (but not with dill tablets) remarkably attenuated LDL-C in HCD-fed rats. 

***Histopathological changes of the hippocampus in HCD-fed rats***

**Figure 1:**
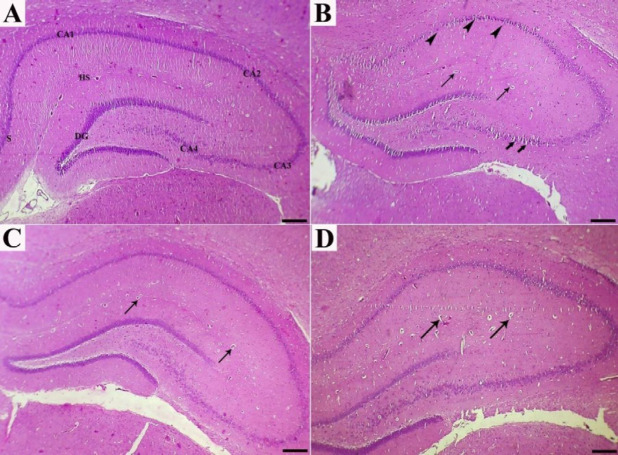
Sections of the hippocampus in experimental rats, as stained by Hematoxylin and Eosin: (a) In normal rats, the hippocampus consists of CA1, CA2, CA3, and CA4 areas; CA1 is continued as subiculum (S). hippocampus sulcus (HS) and dentate gyrus (DG) are narrow. (b) cholesterol-rich diet rats showed prominent reduction in pyramidal cells accompanied by presence of a few shrunken degenerated cells (arrowheads) and vascularization (thin arrows). Slight spongiosis (vacuolations) was also observed in the CA4 area (thick arrows). (c and d) High-cholesterol diet-fed rats treated with dill tablets and aqueous extract of basil showed a better organization in the hippocampus layers and decreased number of capillaries (arrows) with moderate gliosis. Bar=500 µm

**Figure 2:**
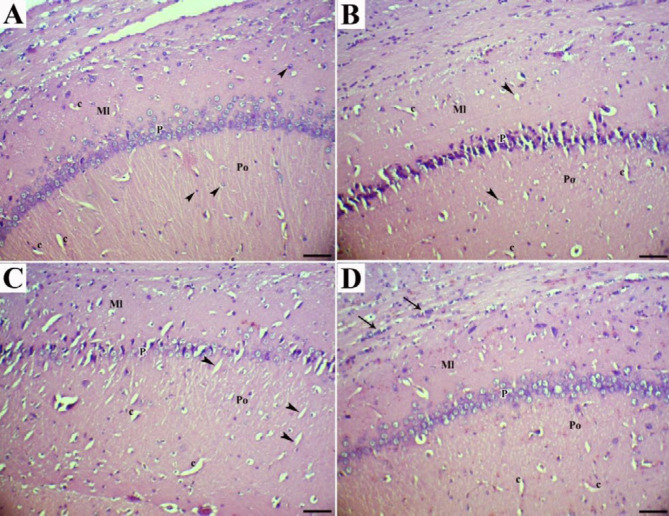
CA1 area of the hippocampus (H&E; Bar=60 µm). (a) normal rats showed molecular (Ml), pyramidal (P), and polymorphic (Po) layers. As shown by arrowheads, the glial cells and capillaries (c) were found irregularly distributed in molecular and polymorphic layers. (b) High cholesterol diet-fed rats showed marked shrinkage in the size of large pyramidal cells, necrotic pyramidal cells with eccentric nuclei dispersed small vacuolations (arrowheads), and vascularization (c). (c) High cholesterol diet-fed rats treated with dill tablets showed less degenerative and necrotic pyramidal cells, moderate gliosis in the molecular layer, and presence of capillaries and small vacuolations in the molecular layer (arrowheads). (d) High-cholesterol diet-fed rats treated with aqueous extract of basil showed pyramidal cells with normal size and shape but clumping of nuclei and neuronal processes and massive gliosis in the molecular layer (arrows)

**Figure 3:**
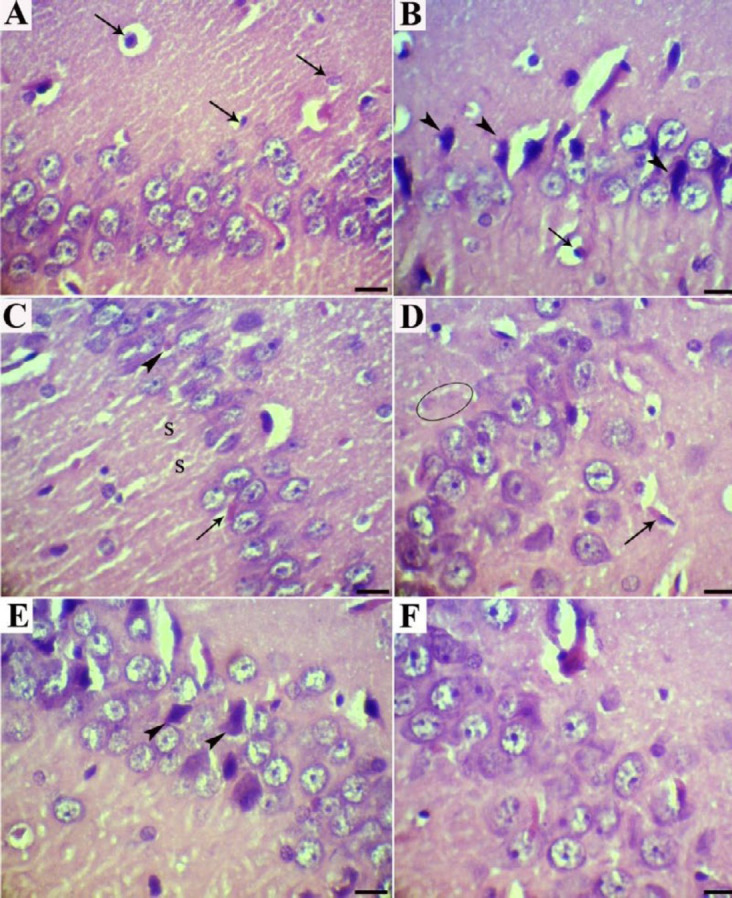
CA1 area of the hippocampus (H&E; Bar=20 µm). (a) Normal rats showed well organized typical pyramidal cells with triangular bodies, the basophilic rim of cytoplasm, large vesicular nuclei, and prominent nucleoli. Different types of neuralgia cells were found irregularly distributed in astrocytes oligodendroglia and microglia (arrows). (b) High-cholesterol diet-fed rats had reduced number of pyramidal cells and showed a few shrunken degenerated cells (arrowheads) as well as apoptotic oligodendroglia (arrow). (c) High-cholesterol diet-fed rats showed extra- (arrow) and intracellular (arrowhead) Hirano's bodies and small vacuoles spread throughout (S). (d) High cholesterol diet-fed rats showed amyloid plaques (circle) and localized apoptosis of oligodendrocytes (arrow). (e) High-cholesterol diet-fed rats treated with dill tablets showed a few pyramidal cells with necrotic changes (arrowheads). (f) High-cholesterol diet-fed rats treated with aqueous extract of basil showed pyramidal cells with normal size and shape, comparable with normal rats

Histologically, the normal hippocampus includes CA1-CA4 areas, each appearing in three polymorphic, pyramidal, and molecular layers. Neuronal processes (axons and dendrites), capillaries, microglia, oligodendroglia and astrocytes, and scattered nerve cells scatter inside molecular and polymorphic layers. The pyramidal nerve cells were observed in large triangular appearance with sizeable vesicular nuclei and projected processes. The subiculum is the outward continuation of the CA1 region (Figures 1A, 2A, and 3A). 

No significant gross pathology was observed in any of the brain tissues at necropsy. Hippocampal tissues of control rats (groups C) showed normal architecture in the hippocampus proper, dentate gyrus, and subiculum. The main histopathologic findings in the rats which received HCD were decreased thickness of the hippocampus, disorganization and shrinking of pyramidal cells, the presence of necrotic pyramidal cells with eccentric nuclei, disintegrated axon, and dispersed small vacuolations, degeneration and vacuolation with enormous neurogliosis and apoptotic oligodendroglia. Moreover, the presence of amyloid plaques, extra- and intracellular actin-rich inclusions and excess glial cells were prominent (Figures 1B, 2B, 3B, 3C, and 3D).

Treatment of the rats with dill tablets and aqueous extract of basil caused an improvement in the histological characterization of the hippocampus in HCD+Dill and HCD+basil groups. In detail, the number of degenerative pyramidal cells, neuralgia cells such as microglia and apoptotic oligodendroglia showed a significant reduction in treated groups. Although pyramidal cells had normal size and shape, clumping of nuclei and neuronal processes, as well as a few intracytoplasmic vacuoles were seen in some cells. The hippocampus layers were more organized and their thickness was almost comparable to the healthy rats. In comparison, the most therapeutic effect was observed in the rats that received basil aqueous extract (Figures 1C, 1D, 2C, 2D, 3E, and 3F).

**Figure 4:**
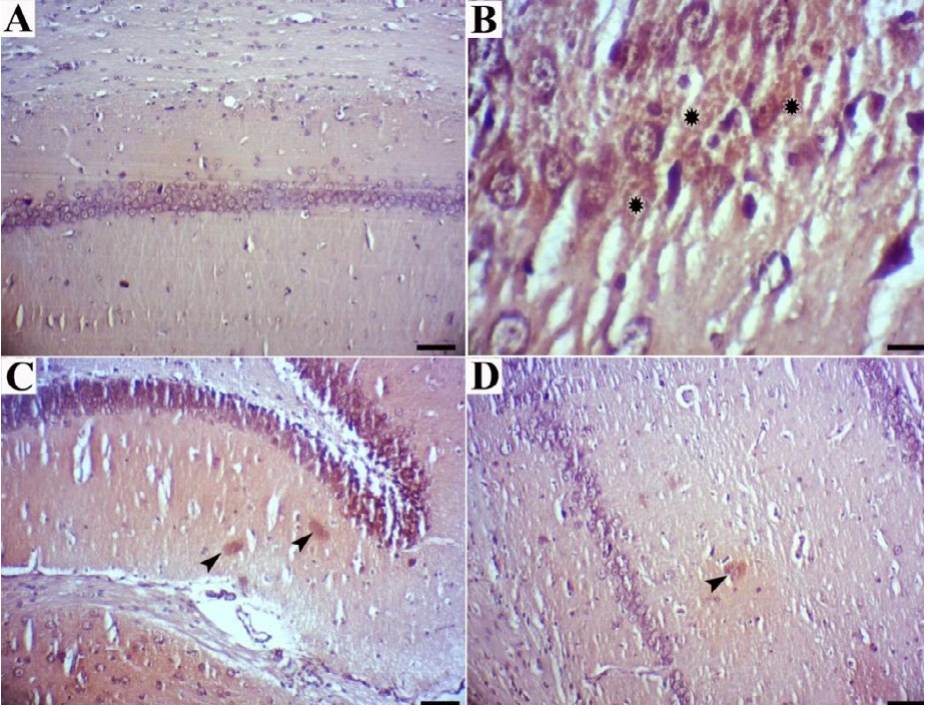
Brain tissues stained with Congo red to visualize Aβ deposits. (a) Normal rats indicated no beta-amyloid deposits (Bar=60 µm). (b) High-cholesterol diet-fed rats showed many Aβ deposits (Bar=20 µm). (c and d) High-cholesterol diet-fed rats treated with dill tablets and aqueous extract of basil showed a few Aβ deposits (Bar=60 µm)

Congo red staining revealed that CA1 neurons in control rats were arranged in an organized fashion, with distinct edges and clear nuclei, without Congo red deposits (Figure 4A), whereas CA1 neurons in rats that received cholesterol-rich diet were surrounded by Congo red deposits in some sections (Figure 4B). In comparison, a few or no stained amyloid deposits were observed in the rats treated with dill tablets or basil extract (Figures 4C and 4D).

***Beneficial effects on MWM performance***

**Figure 5:**
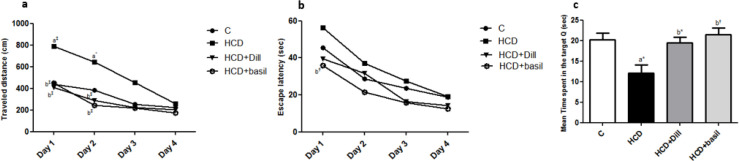
Results of Morris Water Maze (MWM). (a) Average traveled distance, and (b) Average latency time to reach the unseen platform for four consecutive training days. (c) Average time spent in the target quadrant (n=7). ^a*^ and ^a‡^ represent *P*<0.05 and *P*<0.001, respectively vs control group and ^b*, b†^, and ^b‡^ show *P*<0.05, *P*<0.01, and *P*<0.001, respectively compared with high-cholesterol diet (HCD) group

Learning and memory experiments showed that rats fed a high-cholesterol diet (HCD group) traveled further distances to find the hidden platform on days 1 and 2 (out of 4 days training) compared with the control rats, as represented in Figure 5a, although the traveled distances in the third and fourth days of training did not differ between groups. Treatment of the rats with dill tablets or basil extract significantly reduced traveled distances on day 1 (*P*<0.001) and day 2 (*P*<0.001) and lowered it to the distances traveled by the untreated control rats. 

Escape latency of training days increased in the HCD group but this enhancement was not statistically significant. In contrast, treated rats spent less time finding the hidden platform so that treatment with basil extract (HCD+basil) significantly ameliorated (*P*<0.01) the escape latency compared with the HCD group and lowered it to the normal level (Figure 5b).

Analysis of the time spent by the rats in the target quadrant on day 5 of the probe trial showed that HCD-fed animals spent less time compared with control rats (*P*<0.05), whereas treatment with dill tablets or aqueous extract of basil normalized the time to the control level (Figure 5c).

***Improvement in passive avoidance learning acquisition and retention***

**Figure 6:**
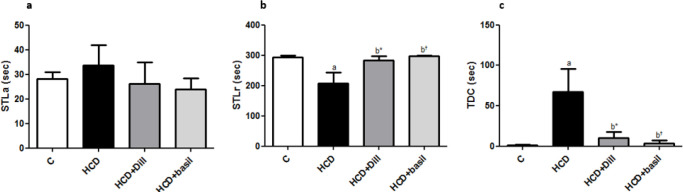
Results of passive avoidance learning (PAL) task among groups (n=7). Mean of step-through latency in (a) acquisition trial (STLa) and (b) retention trial (STLr) as well as time spent in the dark compartment in (c) the retention trial (TDC). ^a^ represents *P*-value less than 0.01 against control group and ^b* ^and ^b†^ show *P*-value less than 0.05 and 0.01, respectively vs high-cholesterol diet (HCD) group

No significant difference was observed in step-through latency in the acquisition trial (STLa) between groups indicating similar and equal exploratory behaviors and native preference of the rats to the dark compartment in all experimental groups (Figure 6a). However, analysis of data from the long-term memory test showed that step-through latency in the retention trial (STLr) was significantly lower in the HCD group compared with healthy rats. Conversely, treatment of the rats with dill tablets or aqueous extract of basil almost completely frustrated the reduction induced in STLr by HCD and normalized the STLr level to that of healthy rats, as shown in Figure 6b. 

A more than 60-fold increase was observed in the mean total time spent in the dark part of the shuttle box in the HCD group whereas untreated control rats and the rats from HCD+Dill and HCD+basil groups spent a little or ignorable time in the dark compartment (Figure 6c). 

***Reduction in hippocampus amyloid β(1-42) content***

**Figure 7:**
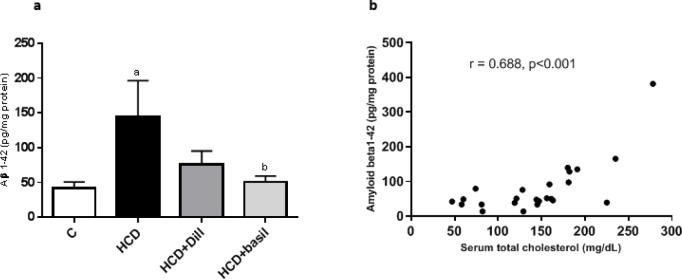
(a) Hippocampus amyloid β(1-42) levels in different experimental groups. (b) Correlation between hippocampus Aβ(1-42) and serum total cholesterol, Pearson correlation coefficient= 0.688, r^2^=0.472, *P*<0.001. ^a^ represents *P*-value less than 0.01 against the control group and ^b ^shows a *P*-value less than 0.05 compared with the HCD group

As shown in Figure 7a, HCD fed rats had a significantly higher level of amyloid β(1-42) in the hippocampus (*P*<0.01) but treatment with dill or basil extract decreased Aβ levels to the amount observed in healthy rats. Basil extract showed 65% greater efficiency than dill in reducing Aβ levels. A positive correlation was also observed (Figure 7b) between serum cholesterol and hippocampus Aβ levels [Pearson correlation (r) = 0.688, r^2^=0.472, *P*<0.001]. 

***Hippocampus fatty acid composition***

The composition of FAs in hippocampus tissues was tabulated in Table 2. Our results showed no significant differences between groups in individual FA contents of the hippocampus and receiving dietary high cholesterol or treatment of the rats with dill tablets or basil extract did not affect FA compositions. 

Serum total cholesterol was found positively correlated with hippocampus n-6 PUFA and total PUFA contents (Table 3) indicating the more serum cholesterol, the more hippocampus n-6 PUFA level. In addition, the hippocampus n-6:n-3 PUFA ratio was found strongly correlated with Aβ(1-42) deposit (Pearson correlation coefficient= 0.588, *P*<0.001), as shown in Table 3. 

***Dill tablets and basil extract affected ABCA1, LXR-α, and LXR-β expression***

**Figure 8:**
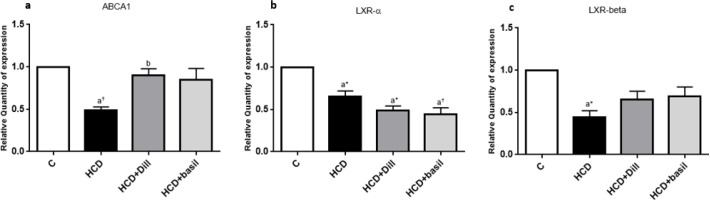
Fold change in hippocampal (n=6) gene expression levels of (a) ABCA1, (b) LXR-α, and (c) LXR-β, as determined by qRT-PCR. The relative expression levels were calculated against β-actin as a housekeeping gene. Data are represented as mean±SEM. ^a*^ and ^a†^ indicate *P*<0.05 and *P*<0.01, respectively against healthy rats and b shows significant difference (*P*<0.05) compared with HCD rats

Expression of the ABCA1 gene was markedly down-regulated in the HCD group (*P*<0.01) compared with healthy rats, while administration of either dill tablets or basil extract corrected the gene expression level up to the value observed in the normal rats (Figure 8a). Consumption of HCD significantly (*P*<0.05) reduced both LXR-α and LXR-β gene expression levels (Figures 8b and 8c). However, while treatment with dill tablets or basil extract was not able to normalize the LXR-α gene expression level, both treatments slightly prevented down-regulation of LXR-β gene expression so that no significant differences were indicated between HCD+Dill or HCD+basil and control groups (Figures 8b and 8c).

***Components of basil aqueous extract***

**Figure 9:**
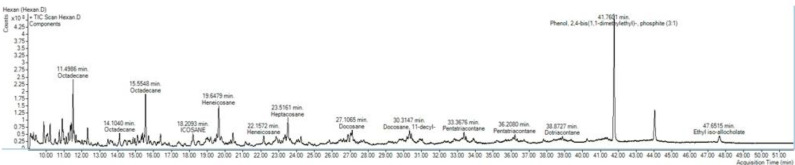
Sample chromatogram of basil aqueous extract, as secondary, extracted by n-hexane and analyzed using the GC-MS/MS technique. Components were identified based on comparison of the spectrum of a given ingredient with the collection of known spectrums available in the National Institute Standard and Technology online database

Components of basil aqueous extract were identified using the GC-MS/MS technique. Totally 70 components were identified in aqueous basil extract using methanol solvent whereas n-hexane, toluene, acetonitrile, and 2-propanol solvents resulted in the identification of 40, 34, 28, and 13 components, respectively (supplementary online resource; Tables 1-5). The major component of n-hexane and toluene extractions was phenol-2,4-bis(1,1-dimethylethyl)-phosphite comprising 16.423% and 31.781% of extracts, respectively (Figure 9). An omega-7 fatty acid, cis-vaccenic acid, was found as a major component (15.251%) of the methanolic extract while palmitoyl monoacylglycerol (37.415%) and pluchidiol (16.085%) were the components with the highest concentrations in acetonitrile extract. Compounds with the highest concentrations in 2-propanol extraction were tricosane (40.111%), 2-Myristynoyl pantetheine (26.363%), and heptacosane (17.011%).

## Discussion

AD affects nearly 40% of the subjects over 85 years old in developed countries and is characterized by deficit cognitive function and everlasting impairment of memory (3). The main histological characteristics of AD are extracellular accumulation of senile amyloid deposits and the presence of intracellular highly phosphorylated tau proteins (14). The prevalence of AD is correlated with the consumption of HCD (1) and it is believed that high blood cholesterol contributes to the pathogenesis of AD by involving in Aβ synthesis, aggregation, neurotoxicity, and elimination from the brain (2). However, despite numerous studies reporting the important function of cholesterol in pathogenesis and development of AD, the underlying mechanisms remain unclear. 

This study showed that HCD induced histopathological changes accompanied with the amyloid-β deposition in the hippocampus of the rats probably through the elevation of serum cholesterol, whereas administration of dill tablets or aqueous extract of basil showed protective effects both on serum lipid profile and hippocampus histology. HCD also affected cognitive functions and reduced learning ability and memory of the rats while these intellectual activities were mainly normalized after administration of dill tablets or aqueous basil extract. HCD did not alter hippocampus FA composition whereas it significantly down-regulated the expression of ABCA1, LXR-α, and LXR-β genes which are involved in cholesterol metabolism. Once again, dill tablets or aqueous extract of basil returned the expression of ABCA1 and LXR-β to normal levels to some extent.

Our results showed that consumption of HCD increased serum total cholesterol and LDL-C levels while administration of dill tablets or basil extract partially prevented these deleterious effects of HCD but did not show any beneficial or adverse effects on HDL-C or TG levels. Dill tablets and aqueous extract of basil reduced serum total cholesterol by 25% and 33%, respectively. Administration of basil extract also lowered LDL-C by 30%. Therefore, this study showed that dill tablets and in particular basil extract are possible candidate agents for adjuvant therapy in hypercholesterolemia. The beneficial hypocholesterolemic effects of basil aqueous extract (15) and dill tablets (10) have previously been reported. These observations indicate that polar components of basil and dill tablets act as hypolipidemic agents and may favorably prevent hyperlipidemia. Hypolipidemic potency of dill might be mediated to some extent, through the inhibition of HMG-CoA reductase, the main regulatory enzyme in biosynthesis of cholesterol (10). As we have previously reported, the mechanism of hypocholesterolemic properties of dill or dill tablets has not been well understood, however, it is believed that dill tablets may exert their hypocholesterolemic effects through regulation of HMG-CoA reductase activity and gene expression. The HMG-CoA reductase inhibitory activity and ability of dill to control endogenous cholesterol biosynthesis are probably attributed to its phenolic, flavonoids, and quercetin components (10). But, the underlying mechanism of the transcriptional regulatory function of the dill tablets remains to be elucidated. 

Our results showed that consumption of HCD induced accumulation of Aβ(142) in hippocampus tissue and a positive correlation was found between serum total cholesterol and hippocampus Aβ deposits showing that the higher serum cholesterol level, the greater hippocampal Aβ(142) content. This observation supports the previously established hypothesis that elevated cholesterol levels may play a role in AD pathology (2, 3). The contribution of elevated cholesterol in AD pathology was later strengthened by our histopathological examinations. Concurrent with the increased serum cholesterol in the HCD group, the presence of amyloid β plaques was also detected by Congo red staining in hippocampal tissues. This accompaniment of elevated cholesterol and deposition of Aβ plaques again was in line with a previous report that showed a higher amyloid β content in brain sections of HCD-fed mice (16). 

Interestingly, deposition of amyloid β plaque was alleviated by treatment of the animals with both dill tablets and aqueous extract of basil, and the histological characterizations of the hippocampus were improved with both types of treatments (HCD+Dill and HCD+basil groups) compared with the untreated HCD group. Histological findings also showed that *O. Basilicum* extract was more therapeutically effective than dill tablets in ameliorating deleterious effects caused by HCD in hippocampal tissue. The higher potency of basil aqueous extract compared with dill tablets in histopathological improvement can be explained by the greater effectiveness of basil in lowering serum cholesterol which was observed earlier in this study. 

Although HCD-fed rats showed increased deposits of Aβ together with histopathological changes in hippocampus tissue that somehow were characteristics of AD, deleterious effects of HCD on cognitive function were also assessed in this study by performing the shuttle box task and MWM to reconfirm the hypothesis of the high cholesterol-AD axis in more depth. Cholesterol-rich diet decreased STLr and increased the time spent in TDC by the HCD group rats. In contrast, consumption of dill tablets and aqueous extract of basil counteracted the negative effects of HCD on memory and learning by normalizing STLr and TDC. It also enhanced spatial memory, as indicated by decreased traveled distance and reduced escape latency in MWM test. Together these data showed that HCD induces AD in the rats whereas dill tablets and aqueous extract of basil exhibit protection against memory loss and learning deficits. 

The deleterious effects of cholesterol-rich diet on the development of AD and memory or learning abilities, as shown in the present study, were somehow expected since HCD-induced deficit in memory/learning or high serum cholesterol-resulted deficits in cognitive function have previously been reported (1, 2). Accordingly, different therapeutic approaches have been considered for attenuating serum cholesterol and therefore for preventing the development and progression of AD such as using inhibitors of HMG-CoA reductase (17) and Acyl-coenzyme A: cholesterol acyl-transferase (ACAT) (18) or dietary supplementation of vitamin E or vitamin C (19). However, since lipid-lowering abilities of *A. Graveolens *L. extract and dill tablets through down-regulation of HMG-CoA reductase gene and inhibition of its activity were observed in our previous study (10), we hypothesized that dill tablets may exhibit a beneficial effect on the HCD-induced AD model by lowering serum cholesterol and attenuating unpleasant aspects of hypercholesterolemia, including alleviation of Aβ deposits and enhancing learning and memory abilities. Also, based on its hypolipidemic activity (9), *O. basilicum *L*.* aqueous extract was also used in this study as a common medicinal herb.

Here we showed for the first time that both dill tablets and aqueous extract of basil mitigated elevation of serum cholesterol, abrogated almost completely HCD-induced hippocampal histopathological alterations, and enhanced memory and learning abilities. It is supposed that these health benefits attributed to dill tablets and aqueous extract of basil are, at least in part, mediated by lowering serum cholesterol. The involvement of oxidative stress in AD has recently been reported (20) and since dill tablets and basil extract have anti-oxidant capacity (8, 21, 22) it is plausible that anti-oxidant activities of these agents might be emerging as another important mechanism of their anti-Alzheimer’s potency. An important implication of the present finding is that dill tablets or basil extract may be used as hypocholesterolemic and anti-oxidant agents and as potential candidate agents in adjuvant therapy of AD. 

The exact mechanisms responsible for attenuation of cognitive deficit by dill tablets or aqueous extract of basil is still unclear. Since high cholesterol level, both increases the production of amyloid-β deposits and promotes the conversion of soluble non-toxic Aβ monomers into an aggregated toxic structure that is critical in the pathogenesis of AD (23), therefore the main possible explanation for dill tablets or aqueous extract of basil advantageously protecting learning and memory is lowering of cholesterol. In addition, as oxidative stress plays key roles in the pathogenesis of AD (24), the anti-oxidant properties of these medicinal plants (due to the phenolic and anthocyanin contents) is another possible mechanism in attenuating deleterious consequences of hypercholesterolemia on brain function and cognition (8, 21, 25). Nevertheless, whether the dill tablets or basil extract can directly protect learning and memory or they indirectly affect cognitive function remains to be elucidated in further studies.

Despite extensive studies about the correlation between cholesterol and cognitive impairment (4) or some evidence about the FA profile of the postmortem brain cortex (26-28), there is no information available in the literature about the FA profile of the hippocampus in AD. Therefore, the current knowledge of the overall changes in FA content in AD is not sufficient. Here, we determined the hippocampus FA composition among different experimental groups. Hippocampus FA composition did not significantly differ between groups, and neither consumption of HCD and nor treatment with dill nor basil affect the FA profile in the hippocampus. Positive correlations were found between serum cholesterol and hippocampus total n-6 PUFA and between hippocampus n-6:n-3 PUFA ratio with hippocampus Aβ(1-42) levels. Collectively, our results suggest that HCD-induced AD is associated with higher serum cholesterol, greater hippocampus Aβ deposition levels, and enhanced hippocampus n-6:n-3 PUFA ratio, although hippocampal FA composition is not altered in AD. However, this conclusion requires further investigations and using other models of AD. 

The correlations between hippocampus n-6:n-3 PUFA, Aβ level, and serum cholesterol which were observed in HCD-induced cognitive impairment may be associated with defects in the metabolism of essential FAs or in transferring components of their parental metabolites to the brain. This finding is not surprising since both n-6 and n-3 types of PUFA are sources for eicosanoid production which may potently influence neuronal function (29). Additionally, although hippocampus FA composition has not been studied so far, elevated levels of both n-6 and n-3 types of PUFA have been reported in other regions of the brain such as a 3-fold increase in 22:4n-6 PUFA level in grey matter in postmortem brain of Alzheimer’s patients (28) or alteration in brain eicosapentaenoic and docosahexaenoic acid levels in mice model of AD (27). However, the exact mechanism underlying observed intriguing correlations remains enigmatic and needs to be delineated. 

Since increased cholesterol level is correlated with enhancement of Aβ (as observed in the present study) and beneficial effects of statins as inhibitors of HMG-CoA reductase (17) in reducing symptoms of AD pathology has already been reported, it is proposed that dysregulation in the expression of the genes encoding proteins for cholesterol-metabolizing and transport pathways may contribute in AD pathology. Determination of ABCA1, LXR-α, and LXR-β gene expression levels showed that high-cholesterol diet significantly down-regulated all three genes in the HCD group but dill tablets or aqueous extract of basil markedly retarded the reduction in gene expression of ABCA1 and LXR-β but not LXR-α. 

ABCA1 plays an important role in AD pathogenesis due to its roles in cholesterol trafficking and metabolization (30). Interestingly, apart from the primary function of ABCA1 as a cholesterol efflux regulatory protein, this protein neutralizes the aggregation capacity of amyloid-β and eases its subsequent removal from the brain (31). It has recently been reported by Yassine *et al*. that subjects with mild cognitive deficits or AD had 30% less ABCA1-mediated cholesterol efflux capacity compared with control subjects (32). Taken together, in this study we showed that HCD down-regulated ABCA1 gene expression and reduced its capacity to eliminate Aβ from the brain. This conclusion was confirmed by our other observation showing that rats from the HCD group had higher Aβ contents. However, it remains puzzling how dill tablets and basil extract may preclude the reduction in ABCA1 gene expression, and further investigations are required to delineate the underlying mechanism involved in transcriptional regulation.

LXR-α and LXR-β gene expression levels were also down-regulated in the present study in the rats fed HCD. In other words, reduced mRNA expression level of LXRs was observed in HCD rats that exhibited AD symptoms such as increased serum cholesterol, accumulation of Aβ protein in the brain, and deficits in cognitive functions. This finding is supported by previous studies that indicate inactivation of the LXRs gene in AD exacerbates amyloid-β deposition (33) whereas agonists of LXR successfully enhance amyloid-β removal and lower Aβ plaque content (33). Co-reduction in gene expression of LXRs and ABCA1, as observed in this study, might be explained in part by the fact that LXRs directly regulate the transcription of an array of genes such as ABCA1 that are responsible for cholesterol trafficking and efflux (33). This is of immense importance since therapeutic agents that activate LXRs or up-regulate LXRs transcription may also modify cholesterol trafficking and Aβ elimination from the brain.

The present study showed the association of increased serum cholesterol with impaired learning and memory. The results also showed beneficial therapeutic effects of dill tablets and aqueous extract of basil in ameliorating unpleasant effects induced by HCD on hippocampus Aβ levels and cognitive functions. However, our results may be considered preliminary at this stage and further investigations involving determination of free and esterified cholesterol in hippocampus tissue, measurement of phosphorylated tau protein, and neuron-specific staining of the hippocampus to detect the severity of neurodegeneration are required to strengthen the findings.

**Table 1:** Bodyweight and serum lipid profile in control, high-cholesterol diet fed rats, and in animals treated with Dill and basil extract

		**Control (C)**	**HCD**			**HCD+Dill**	**HCD+basil**
	** Initial weight**	190.8± 2.45		191.1±2.78	192.6±2.49		196.6±1.98
	** Final weight**	337.2±10.78		358.7±12.87	356.7±15.5		375.6±7.45
**Start day**							
	**TC** (mg/dl) **TG** (mg/dl) **LDL-C** (mg/dl) **HDL-C** (mg/dl)	81.0±2.17 89.9±4.66 29.32±2.80 33.7±2.67		78.6±4.80 91.3±5.75 27.24±5.78 33.1±2.28	76.3±2.61 87.6±7.86 23.88±3.56 34.9±1.68		76.8±1.57 86.3±4.79 23.94±1.95 35.6±1.79
**End day**							
	**TC** (mg/dl) **TG** (mg/dl) **LDL-C** (mg/dl) **HDL-C** (mg/dl)	67.00±5.77 66.33±1.83 19.00±1.39 32.50±0.99		210.33±17.72^a†^ 61.33±5.60 113.67±10.8^a†^ 27.50±2.20	157.8±10.67^a†,b*^ 51.50±3.29 109.00±9.06^a†^ 25.83±3.15		139.17±6.45^a*,b†^ 57.00±3.95 79.33±4.55^a†,b*^ 27.00±3.09

Values are expressed as mean±SEM. ^a*^ *P*<0.01 and ^a†^ *P*<0.001 vs control group ^b*^ *P*<0.05 and b† *P*<0.01 vs HCD group

HCD: high-cholesterol diet

HCD: high-cholesterol diet

**Table 2:** Hippocampus fatty acid composition in control, high-cholesterol diet fed rats, and in animals treated with Dill and basil extract

	**C**	**HCD**	**HCD+Dill**	**HCD+basil**
**C14:0**	0.80 ± 0.08	2.30 ± 0.84	2.46 ± 0.72	2.45 ± 0.78
**C16:0**	22.35 ± 1.73	20.32 ± 1.65	21.52 ± 0.95	21.34 ± 3.10
**C18:0**	24.93 ± 1.98	21.90 ± 1.44	19.80 ± 1.54	18.98 ± 2.51
**C18:1n-9**	6.25 ± 1.85	7.99 ± 1.73	10.71 ± 1.33	8.39 ± 2.19
**C18:2 (LA)**	7.95 ± 1.04	9.68 ± 1.41	7.64 ± 0.78	6.40 ± 0.88
**C22:0**	6.57 ± 1.75	6.76 ± 1.78	3.45 ± 2.05	5.58 ± 1.86
**C22:1**	1.39 ± 0.30	1.75 ± 0.49	2.16 ± 1.09	1.83 ± 0.45
**C20:4 (5,8,11,14)**	6.47 ± 1.99	3.30 ± 1.43	6.90 ± 2.16	4.86 ± 3.88
**C22:6 (DHA)**	4.05 ± 1.97	5.36 ± 1.89	8.42 ± 0.02	4.24 ± 1.51
**Total SFA**	52.83 ± 4.33	48.83 ± 3.62	45.67 ± 3.12	45.17 ± 5.06
**Total MUFA**	6.83 ± 1.66	7.50 ± 1.60	12.17 ± 1.85	9.00 ± 1.82
**Total n-6 PUFA**	12.60 ± 1.17	14.17 ± 1.62	12.67 ± 1.58	9.17 ± 1.44
**Total n-3 PUFA**	4.07 ± 1.97	5.36 ± 1.89	8.34 ± 0.09	4.24 ± 1.51
**Total PUFA**	15.31 ± 2.51	17.74 ± 2.57	19.62 ± 2.31	13.40 ± 1.39
**Total UFA**	22.15 ± 9.47	25.24 ± 8.80	31.78 ± 8.37	22.40 ± 7.22
**n-6:n:3 PUFA Ratio**	6.30 ± 2.72	16.07 ± 13.68	1.56 ± 0.23	7.48 ± 3.46
**UFA:SFA Ratio**	0.47 ± 0.13	0.55 ± 0.10	0.72 ± 0.09	0.56 ± 0.11
**PUFA:SFA Ratio**	0.32 ± 0.08	0.38 ± 0.07	0.44 ± 0.05	0.32 ± 0.05

HCD: high-cholesterol diet

**Table 3:** Correlation between serum total cholesterol (TC) and Aβ(1-42) with hippocampus fatty acid composition in high-cholesterol diet fed rats

**Parameters **		**r** ^2^	**Pearson correlation**	***P*** **-value**
**Serum total ** **cholesterol**	**Total SFA**	0.006	-0.078	0.326
	**Total MUFA**	0.001	-0.038	0.413
	**Total n-6 PUFA**	**0.14**	**0.371**	0.013*
	**Total n-3 PUFA**	0.013	0.110	0.277
	**Total PUFA**	**0.106**	**0.316**	0.030*
	**Total UFA**	0.044	0.205	0.115
	**n-6:n-3 PUFA Ratio**	0.061	0.243	0.094
	**UFA:SFA Ratio**	0.025	0.136	0.214
				
**Hippocampus** **Aβ(1-42)**	**Total SFA**	0.015	0.124	0.236
	**Total MUFA**	0.002	-0.44	0.400
	**Total n-6 PUFA**	0.044	0.209	0.111
	**Total n-3 PUFA**	0.019	-0.136	0.232
	**Total PUFA**	0.011	0.108	0.266
	**Total UFA**	0.003	0.054	0.376
	**n-6:n-3 PUFA Ratio**	**0.351**	**0.588**	0.000*
	**UFA:SFA Ratio**	0.001	-0.021	0.452

## Conclusion

Dill tablets and aqueous extract of basil reduced serum cholesterol levels, decreased hippocampus Aβ deposit, and enhanced learning and memory abilities. The effectuality of aqueous extract of basil in attenuating HCD-induced cognitive deficits was greater than dill tablets. Therefore, dill tablets and basil extract might be beneficial agents in adjuvant therapy of AD.
